# Universal Dental Adhesives Containing Zinc Oxide and Copper Nanoparticles Improve Interface on Caries‐Affected Dentin After 2 Years: In Vitro Study

**DOI:** 10.1111/jerd.70095

**Published:** 2025-12-30

**Authors:** Mario Felipe Gutiérrez, Romina Aliaga‐Gálvez, Romina Ñaupari‐Villasante, Eduardo Fernández, Alessandro D. Loguercio

**Affiliations:** ^1^ Centro de Investigación e Innovación Biomédica (CIIB) Universidad de los Andes, Chile. Facultad de Odontología Santiago Chile; ^2^ Department of Restorative Dentistry, Faculty of Dentistry University of Chile Santiago Chile; ^3^ Department of Restorative Dentistry, School of Dentistry State University of Ponta Grossa Ponta Grossa Brazil; ^4^ Instituto de Ciencias Biomédicas, Universidad Autónoma de Chile Santiago Chile

**Keywords:** carious dentin, copper, microtensile bond strength, nanoleakage, nanoparticles, universal adhesive system, zinc oxide

## Abstract

**Objective:**

To evaluate the effect of adding zinc oxide and copper nanoparticles (ZnO/CuNp) into universal adhesives (UAs) on resin–dentin microtensile bond strength (μTBS), nanoleakage (NL), and presence of ZnO/Cu within the hybrid layer on caries‐affected dentin, both at baseline (T0) and after 2 years of water storage (T2).

**Materials and Methods:**

ZnO/CuNp (0% [control]; 5/0.1 and 5/0.2 wt%) were added into Prime&Bond Active (PBA) and Ambar Universal (AMB). Ninety‐six extracted human third molars were used. After microbiological induction of caries‐affected dentin, UAs were applied in both etch‐and‐rinse and self‐etch modes, followed by composite build‐up. Specimens were sectioned to obtain resin–caries‐affected dentin bonded sticks for μTBS, NL, and chemical characterization of the hybrid layer for Cu and Zn detection at T0 and T2. Data were statistically analyzed (*α* = 0.05).

**Results:**

ZnO/CuNp had no effect on μTBS at T0 (*p* > 0.05) but improved μTBS at T2, compared to control (*p* < 0.05). Both UAs showed a significant decrease in μTBS over time (*p* < 0.05), except PBA with ZnO/CuNp in etch‐and‐rinse mode. NL was significantly lower in ZnO/CuNp‐containing UAs at both time points (*p* < 0.05), though all groups showed increased NL over time. ZnO/CuNp remained detectable in the hybrid layer at T2.

**Conclusions:**

Adding ZnO/CuNp to UAs decreased adhesive interface degradation on caries‐affected dentin, likely due to their presence within the hybrid layer even after 2 years.

**Clinical Significance:**

ZnO/Cu nanoparticles into UAs could enhance the stability of adhesive restorations on caries‐affected dentin by reducing interface degradation.

## 1 Introduction

Caries‐affected dentin is a common substrate for adhesive restorations in clinical practice [1, 2]. However, this substrate is highly unfavorable for bonding with resin composite restorations [3, 4], mainly after partial caries removal [5, 6]. This poor bonding performance is mainly due to the altered cross‐striated pattern of the collagen fibril organic matrix, the presence of non‐collagenous proteins, increased porosity, and reduced mineral content [7, 8].

These characteristics mean that this substrate has reduced mechanical properties, as well as high humidity [9]. Furthermore, due to intermittent pH fluctuations produced during caries progression, we can observe a higher rate of different collagenolytic enzymes activation [10], promoting collagen degradation [11, 12]. Thus, the adhesive procedure on caries‐affected dentin poses a significant challenge for clinicians. The development of materials with antibacterial activity, collagenolytic enzyme inhibition, and collagen cross‐linking capabilities is essential to enhance the long‐term durability of the resin–dentin interface on caries‐affected dentin, while preserving the mechanical properties of the restorative material [13].

As we know, metallic ions, even in lower concentrations, maintain potent antimicrobial activity. Among them, copper (CuNp) and zinc oxide (ZnONp) nanoparticles have been highlighted as promising antimicrobial agents when added to adhesive systems [14, 15, 16, 17, 18, 19, 20]. When were incorporated into an adhesive system, CuNps have been shown to improve bonding properties to sound dentin and reduce degradation of the adhesive interface even after 4 years of water storage [16], without compromising clinical performance in either the short [21] or long term [22]. On the other hand, the addition of ZnONp into an adhesive system increases its antimicrobial properties and preserves the bonding to sound dentin after 6 months [14], reducing collagen degradation [23, 24, 25]. In both cases, the incorporation of CuNp or ZnONp did not reduce the mechanical properties of the adhesive nor affect its immediate bond strength to dentin [14, 26, 27, 28]. It is worth noting that both ions exhibit potent inhibitory effects on dentin enzymes and may also enhance the strength of the collagen network [29, 30, 31], by promoting additional cross‐linking [32].

To the best of our knowledge, only two studies have reported that the combined incorporation of zinc oxide and copper nanoparticles (ZnO/CuNp) into adhesive system is a feasible strategy, offering antimicrobial activity against *S. mutans*, while also simultaneously enhancing the integrity of the hybrid layer in both sound [33] and caries‐affected dentin in the immediate term [34]. However, no long‐term studies have evaluated whether the incorporation of ZnO/CuNp into adhesive systems can sustain the preservation of the hybrid layer on caries‐affected dentin. Moreover, these ions have been detected within the hybrid layer even after extended periods of water storage. Therefore, this in vitro study aimed at investigating the effect of addition of ZnO/CuNp in different concentrations into two commercial universal adhesives (UA) systems on the microtensile bond strength (μTBS), nanoleakage (NL), and presence of these ions into hybrid layer in the resin–caries‐affected dentin interface after 2 years of water storage. Thus, the following research hypotheses were tested: (1) there would be significant differences in the μTBS and NL when two UAs with different ZnO/CuNp concentrations were used (5/01% and 5/02%) at the baseline and after aging, compared to UAs without ZnO/CuNp (control); (2) there would be significant difference in the μTBS and NL between baseline and after 2 years of water storage for UAs tested; and (3) there would still be presence of zinc and copper within the hybrid layer of UAs with different ZnO/CuNp concentrations after 2 years.

## 2 Materials and Methods

### 2.1 Formulation of the Experimental Adhesives

As previously described [14, 17, 18, 35], experimental UAs were prepared using Prime&Bond Active (Dentsply‐Sirona, Konstanz, Baden‐Württemberg, Germany) and Ambar Universal (FGM Dental Group, Joinville, SC, Brazil). Six formulations were obtained by incorporating ZnONp (99.8% pure, SkySpring Nanomaterials, Houston, TX, USA) and CuNp (99.9% pure, SkySpring Nanomaterials) at different concentrations (Zn/Cu wt%: 0% [control], 5/0.1%, and 5/0.2%). The incorporation was performed in a dark room using a motorized stirrer for 1 min, as previously reported [17].

### 2.2 Teeth Preparation and Bonding Procedures

Ninety‐six caries‐free human third molars (18–35 years old) were collected after approval of the Ethics Committee of State University of Ponta Grossa (PR, Brazil; protocol 2.399.496). Teeth were disinfected in 0.5% chloramine, stored in distilled water and used within 3 months. A flat dentin surface was prepared by removing the occlusal enamel with 180‐grit SiC paper (Buehler Ltd., Lake Bluff, IL, USA).

### 2.3 Microbiological Caries Induction

This method was similar to previously described [36]. Briefly, all tooth surfaces except the occlusal were coated with epoxy resin (Araldite, Brascola, São Bernardo do Campo, SP, Brazil) and nail varnish (Colorama Maybelline, São Paulo, SP, Brazil). Sterilization was performed in a steam autoclave (Phoenix Brasileira, Araraquara, SP, Brazil) at 121°C for 15 min. Each tooth specimen was then immersed in 8 mL of artificial caries solution containing brain heart infusion, yeast extract, sucrose, and glucose in 250 mL of distilled water and 100 μL of *S. mutans* (ATCC 25175) at pH ~4.0. The samples were incubated anaerobically (5% CO_2_, 37°C), and the medium was renewed every 48 h. After 14 days, the specimens were re‐sterilized and rinsed in deionized water [36].

### 2.4 Bonding Procedures

The bonding procedures followed the methodology of our previous studies [34, 37], with minor modifications. Briefly, the surrounding enamel was removed with a diamond bur #4137 (KG Sorensen, Barueri, SP, Brazil), and the occlusal dentin was polished with 600‐grit SiC paper for 30 s to standardize the smear layer and simulated caries‐affected dentin [34].

All UAs (with and without ZnO/CuNp) were applied in etch‐and‐rinse (ER) or self‐etch (SE) mode according to manufacturer instructions (Table 1) (*n* = 8 per group) [37]. For the ER mode, dentin was etched with 37% phosphoric acid (Condac, FGM Dental Group, Joinville, SC, Brazil) for 15 s, rinsed, and gently dried. Composite resin buildups (Opallis, FGM Dental Group, Joinville, SC, Brazil) were placed in three increments of 1 mm, each light‐cured for 40 s (High Power mode, 1200 mW/cm^2^, VALO, Ultradent Products, South Jordan, UT, USA). All procedures were performed by a single operator under controlled conditions. Group size followed Academy of Dental Materials recommendations [37].

**TABLE 1:** Universal adhesive system (batch number), composition (*), and application mode.

Universal adhesive system (manufacturer, batch number) and pH	Composition (*)	Etch‐and‐rinse mode	Self‐etch mode
Prime&Bond Active (PBA—Dentsply‐Sirona, Konstanz, Baden‐Württemberg, Germany) (1703000452) pH = ~2.5	Phosphoric acid modified acrylate resin, multifunctional acrylate, bifunctional acrylate, acidic acrylate, isopropanol, water, initiator, Stabilizer (10‐MDP and PENTA)	Apply phosphoric acid for 15 s. Remove gel with vigorous water spray and rinse conditioned areas thoroughly for 15 s. Remove rinsing water completely by blowing gently with an air syringe or blot dry. Do not desiccate dentin. Apply adhesive to completely wet the surfaces to be treated. If necessary rewet applicator tip. Avoid pooling of the adhesive. Keep the adhesive slightly agitated for 20 s. Disperse adhesive and remove solvent with clean, dry air from an air‐water syringe. Treat every surface with a moderate air flow for at least 5 s until a glossy and uniform layer results. Light cure for 10 s at 1200 mW/cm^2^.	Apply adhesive to completely wet the surfaces to be treated. If necessary rewet applicator tip. Avoid pooling of the adhesive. Keep the adhesive slightly agitated for 20 s. Disperse adhesive and remove solvent with clean, dry air from an air‐water syringe. Treat every surface with a moderate air flow for at least 5 s until a glossy and uniform layer results. Light cure for 10 s at 1200 mW/cm^2^.
Ambar Universal (AMU—FGM Dental Group, Joinville, Santa Catarina, Brazil) (310516) pH = 2.6–3.0	10‐MDP, methacrylic monomers, photoinitiators, coinitiators, stabilizers, silica nanoparticles, and ethanol	Apply phosphoric acid for 15 s. Wash the surface with plenty of water and dry the cavity so that the dentin does not get dehydrated, but without the accumulation of water on the surface. Apply a first layer vigorously rubbing the adhesive with the micro applicator for 10 s. Next, apply a second layer of adhesive for 10 s, spreading the product. Evaporate excess solvent by thoroughly air‐drying with an air syringe for 10 s Light cure for 10 s at 1200 mW/cm^2^.	Apply a first layer vigorously rubbing the adhesive with the micro applicator for 10 s. Next, apply a second layer of adhesive for 10 s, spreading the product. Evaporate excess solvent by thoroughly air‐drying with an air syringe for 10 s. Light cure for 10 s at 1200 mW/cm^2^.

*Note*: (*) 10‐MDP = methacryloyloxydecyl dihydrogen phosphate; PENTA = dipentaerythritol penta acrylate monophosphate.

After 24 h storage in distilled water at 37°C specimens were sectioned using a diamond saw (IsoMet 1000; Buehler, Lake Bluff, USA). Following previous protocols [38, 39], resin‐dentin bonded slices (~1.2 mm) were obtained for NL test, while resin‐dentin bonded sticks (~0.8 mm^2^) were prepared for μTBS and energy‐dispersive X‐ray (EDX) analysis. Premature failures (PF) were recorded and excluded from bond strength calculation. Specimens were tested either immediately or after 2 years of water storage (37°C, pH monitored monthly). For each tooth: Two slices were used for NL; two sticks were used for EDX analysis, and remaining sticks were subjected to μTBS.

### 2.5 Microtensile Bond Strength Testing

The μTBS test followed the procedure described previously [38, 39]. Briefly, each stick was attached to a jig with cyanoacrylate resin (IC‐Gel, bSi, Atascadero, CA, USA) and tested under tensile force at 0.5 mm/min (Kratos, São Paulo, SP, Brazil). Failure mode was classified under an optical stereomicroscope (40×; SZH‐131, Olympus; Tokyo, Japan) as cohesive in dentin, cohesive in resin, adhesive, or mixed [38, 39]. Premature failures were also recorded.

### 2.6 Nanoleakage Evaluation

NL evaluation was performed as described previously [17]. In summary, specimens were coated with nail varnish, immersed in ammoniacal silver nitrate solution for 24 h in the dark, rinsed, and developed under fluorescent light. Specimens were mounted on aluminum stubs, polished with 1000‐, 1500‐, 2000‐, and 2500‐grit SiC paper and 1 and 0.25 μm diamond paste (Buehler, Lake Bluff, IL, USA). Then, they were ultrasonically cleaned, air dried, dehydrated for 24 h in a container with silica gel, and gold sputter coated (MED 010, Balzers Union, Balzers, Liechtenstein). The interfaces were examined using a scanning electron microscope in backscattered mode at 12 kV and 1000× magnification (VEGA 3 TESCAN, Shimadzu, Tokyo, Japan).

In a way to standardize image acquisition, five pictures were taken of each specimen. The first picture was taken in the center of the resin–dentin bonded slice. The other four pictures were taken 0.3 and 0.6 mm to the left and right of the first one. Two resin–dentin bonded slice specimens for each tooth and at each storage period were used for NL evaluation, totalizing 160 images per group. A technician who was blinded to the experimental conditions under evaluation took them all. The relative percentage of NL within the adhesive and hybrid layer areas was measured in all pictures using the public domain ImageJ software, a Java‐based image processing software package developed at the National Institutes of Health (NIH; USA) [40].

### 2.7 Identification of Zinc Oxide and Copper Within the Hybrid Layers by Energy‐Dispersive X‐Ray

Two sticks per each tooth were analyzed in a field emission scanning electron microscope (MIRA 3 TESCAN, Shimadzu, Tokyo, Japan) equipped with EDX, at 2000× magnification, for identification of the presence of zinc oxide and copper within the hybrid layer. The bonding area was observed and the analysis was focused on the middle of the hybrid layer. Five pictures were taken of each specimen. The first picture was taken in the center of the resin–dentin bonded stick, within the hybrid layer. The other four pictures were taken 0.3 and 0.6 mm to the left and right of the first one. One of these images, randomly determined, per resin–caries‐affected dentin bonded stick was examined.

### 2.8 Statistical Analysis

The data for μTBS (MPa) and NL (%) were first analyzed using the Kolmogorov–Smirnov test to assess whether the data followed a normal distribution, as well as Barlett's test to verify the equality of variances. Data normality and homogeneity of variances were confirmed (*p* = 0.32 and *p* = 0.27, respectively). After these assumptions were met, the μTBS (MPa) and NL (%) results for each UA were analyzed using two‐way ANOVA (ZnO/CuNp group vs. adhesive strategy) followed by paired *t*‐tests to assess the effect of storage time. Tukey's post hoc test was used for pair‐wise comparisons (*α* = 0.05) using the Statistica for Windows software (StatSoft, Tulsa, OK, USA). The identification of zinc and copper within the hybrid layers by EDX was only qualitatively evaluated.

## 3 Results

### 3.1 Microtensile Bond Strength

For Prime&Bond Active, no significant differences were observed in μTBS among all groups, in both adhesive strategies, at the baseline (Table 2; *p* > 0.45). After 2 years, it was observed that the addition of ZnO/CuNp showed higher μTBS values than control, in both adhesive strategies (Table 2; *p* < 0.003). No significant difference in the μTBS between baseline and after 2 years was observed for 5/0.1 and 5/0.2 groups, in ER strategy (Table 2; *p* > 0.05). The predominant failure mode was adhesive, regardless of adhesive strategy and time (Table 3).

**TABLE 2:** Means and standard deviations of microtensile bond strength (MPa) obtained in each experimental condition with Prime&Bond Active.

Time	Etch‐and‐rinse			Self‐etch		
	0 (control)	5/0.1	5/0.2	0 (control)	5/0.1	5/0.2
Baseline	27.2 ± 6.3 A	27.7 ± 6.5 A	27.9 ± 4.5 A	29.8 ± 2.1 A	30.2 ± 5.3 A	30.7 ± 4.3 A
2 years	15.6 ± 1.8 b	23.5 ± 2.5 a	23.0 ± 2.1 a	15.7 ± 1.9 b	21.6 ± 1.7 a	21.8 ± 1.1 a
*p* value	0.03*	0.35	0.09	0.0003*	0.02*	0.02*

*Note*: Comparisons are valid only within same row. Means identified with the same capital or lowercase letter are statistically similar (Tukey's test, *p* ≥ 0.05). Comparisons are valid only within same column. Asterisk sign (*) identify means statistically different (*tt*‐test, *p* < 0.05).

**TABLE 3:** Fracture pattern, premature failures, and number of resin–dentin bonded sticks (*n*) tested in the microtensile bond strength test in each experimental condition with Prime&Bond Active (*).

ZnO/Cu concentration (%)	Baseline											
	Etch‐and‐rinse						Self‐etch					
	Premature failure	*n*	Fracture pattern				Premature failure	*n*	Fracture pattern			
			CD	CR	A	M			CD	CR	A	M
0 (control)	2	40	0	0	38	0	1	40	0	0	39	0
5/0.1	1	40	0	0	39	0	1	40	0	0	39	0
5/0.2	0	40	0	1	39	0	0	40	0	0	39	1
	2 years											
0 (control)	3	40	3	0	34	0	4	40	4	0	32	0
5/0.1	1	40	0	0	37	2	1	40	1	0	36	2
5/0.2	1	40	1	0	37	1	1	40	0	0	37	2

*Note*: (*) Classification of fracture pattern: CD—cohesive dentin; CR—cohesive in resin; A—adhesive; and M—mixed.

For Ambar Universal, at baseline, no significant differences were observed in the μTBS among all groups, regardless of adhesive strategy (Table 4; *p* > 0.05). A significant difference between adhesive strategies was observed only in the control group, where the SE strategy showed lower μTBS values than the ER strategy (Table 3; *p* < 0.01). After 2 years, it was observed that the addition of ZnO/CuNp showed higher μTBS values than the control, in both adhesive strategies (Table 4; *p* < 0.01). A significant decrease in μTBS was observed after 2 years for all groups, in both the ER strategy and the SE strategy (Table 4; *p* < 0.02). The predominant failure mode was adhesive, regardless of adhesive strategy, and time (Table 5).

**TABLE 4:** Means and standard deviations of microtensile bond strength (MPa) obtained in each experimental condition with Ambar Universal (*).

Time	Etch‐and‐rinse			Self‐etch		
	0 (control)	5/0.1	5/0.2	0 (control)	5/0.1	5/0.2
Baseline	29.8 ± 2.9 A	29.4 ± 3.2 A	32.6 ± 4.7 A	19.7 ± 5.4 B	25.2 ± 2.7 AB	25.6 ± 1.8 AB
2 years	15.5 ± 1.6 b	22.3 ± 1.3 a	22.6 ± 2.2 a	12.9 ± 0.9 b	19.5 ± 2.2 a	19.8 ± 1.0 a
*p* value	0.01*	0.04*	0.03*	0.02*	0.006*	0.006*

*Note*: Comparisons are valid only within same row. Means identified with the same capital or lowercase letter are statistically similar (Tukey's test, *p* ≥ 0.05). Comparisons are valid only within same column. Asterisk sign (*) identify means statistically different (‐test, *p* < 0.05).

**TABLE 5:** Fracture pattern, pre‐tested failures and number of resin–dentin bonded sticks (*n*) tested in the microtensile bond strength test in each experimental condition with Ambar Universal (*).

ZnO/Cu concentration (%)	Baseline											
	Etch‐and‐rinse						Self‐etch					
	Premature failure	*n*	Fracture pattern				Premature failure	*n*	Fracture pattern			
			CD	CR	A	M			CD	CR	A	M
0 (control)	2	40	1	0	37	0	2	40	0	0	39	1
5/0.1	1	40	1	0	37	1	1	40	0	1	39	0
5/0.2	1	40	0	0	37	2	0	40	0	1	38	1
	2 years											
0 (control)	3	40	4	0	32	1	4	40	4	0	32	0
5/0.1	2	40	1	0	37	0	2	40	2	0	35	1
5/0.2	0	40	1	0	38	1	1	40	1	0	38	0

*Note*: (*) Classification of fracture pattern: CD—cohesive dentin; CR—cohesive in resin; A—adhesive; and M—mixed.

### 3.2 Nanoleakage Evaluation

**FIGURE 1:**
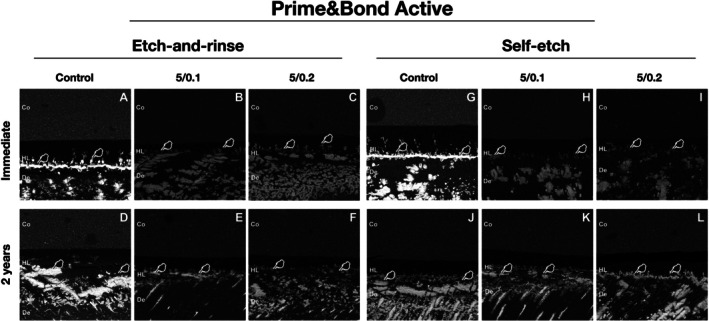
Representative back‐scattering SEM images of the resin–dentin interfaces according to the different experimental conditions using Prime&Bond Active adhesive. In the ZnO/CuNp‐containing adhesive groups (B and C and E and F for etch‐and‐rinse mode; H and I and K and L for self‐etch mode) we can see lower silver nitrate deposition than the control groups (A and D for etch‐and‐rinse mode; G and J for self‐etch mode) (white stains indicated by white pointer). Co = composite; De = dentin; HL = hybrid layer.

For Prime&Bond Active, it was observed that the addition of ZnO/CuNp showed lower NL values than control, in both adhesive strategies, at the baseline and after 2 years (Figure 1 and Table 6; *p* < 0.0008). A significant increase in the NL values after 2 years was observed for all groups, regardless of adhesive strategy (Figure 1 and Table 6; *p* < 0.0005).

**TABLE 6:** Means and standard deviations of nanoleakage (%) obtained in each experimental condition with Prime&Bond Active (*).

Time	Etch‐and‐rinse			Self‐etch		
	0 (control)	5/0.1	5/0.2	0 (control)	5/0.1	5/0.2
Baseline	13.3 ± 2.8 B	8.0 ± 1.7 A	7.3 ± 1.9 A	14.6 ± 2.2 B	8.4 ± 1.5 A	7.0 ± 1.6 A
2 years	32.9 ± 3.8 b	15.9 ± 3.7 a	13.1 ± 3.0 a	35.3 ± 3.6 b	17.5 ± 2.7 a	16.8 ± 2.1 a
*p* value	< 0.0001*	0.009*	0.02*	< 0.0001*	0.0006*	0.0002*

*Note*: Comparisons are valid only within same row. Means identified with the same capital or lowercase letter are statistically similar (Tukey's test, *p* ≥ 0.05). Comparisons are valid only within same column. Asterisk sign (*) identify means statistically different (*tt*‐test, *p* < 0.05).

**FIGURE 2:**
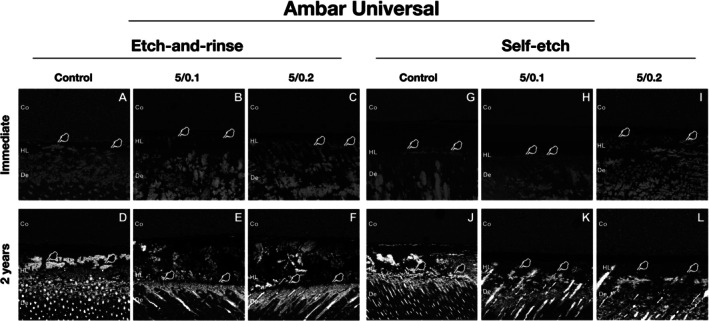
Representative back‐scattering SEM images of the resin–caries‐affected dentin interfaces according to the different experimental conditions using Ambar Universal adhesive. In the ZnO/CuNp‐containing adhesive groups (B and C and E and F for etch‐and‐rinse mode; H and I and K and L for self‐etch mode) we can see lower silver nitrate deposition than the control groups (A and D for etch‐and‐rinse mode; G and J for self‐etch mode) (white stains indicated by white pointer). Observe, also, the lower amount of silver nitrate deposition after 2 years for 5/0.2 group in the self‐etch mode when compared to etch‐and‐rinse mode (I and L). Co = composite; De = dentin; HL = hybrid layer.

For Ambar Universal, at baseline, it was observed that the addition of ZnO/CuNp showed lower NL values than control, in both adhesive strategies (Figure 2 and Table 7; *p* < 0.0004). A significant difference between adhesive strategies was observed only in the control group, with the ER strategy showing higher NL values than the SE strategy (Figure 2 and Table 7; *p* = 0.0003). After 2 years, it was observed that the addition of ZnO/CuNp showed lower NL values than control, in both adhesive strategies (Figure 2 and Table 7; *p* < 0.02). A significant difference between adhesive strategies was observed only in the 5/0.2 group, with the SE strategy showing lower NL values than the ER strategy (Figure 2 and Table 7; *p* = 0.03). A significant increase in the NL values after 2 years was observed for all groups, regardless of adhesive strategy (Figure 2 and Table 7; *p* < 0.001).

**TABLE 7:** Means and standard deviations of nanoleakage (%) obtained in each experimental condition with Ambar Universal (*).

Time	Etch‐and‐rinse			Self‐etch		
	0 (control)	5/0.1	5/0.2	0 (control)	5/0.1	5/0.2
Baseline	13.7 ± 4.3 C	3.9 ± 1.4 AB	3.4 ± 1.4 A	7.4 ± 2.8 B	3.2 ± 1.2 A	3.0 ± 0.6 A
2 years	38.7 ± 4.5 c	21.1 ± 1.9 b	17.7 ± 2.6 b	37.2 ± 2.4 b	17.9 ± 2.3 b	13.5 ± 1.9 a
*p* value	< 0.0001*	< 0.0001*	< 0.0001*	< 0.0001*	< 0.0001*	< 0.0001*

*Note*: Comparisons are valid only within same row. Means identified with the same capital or lowercase letter are statistically similar (Tukey's test, *p* ≥ 0.05). Comparisons are valid only within same column. Asterisk sign (*) identify means statistically different. (‐test, *p* < 0.05).

### 3.3 Identification of Zinc and Copper Within the Hybrid Layers by EDX

**FIGURE 3:**
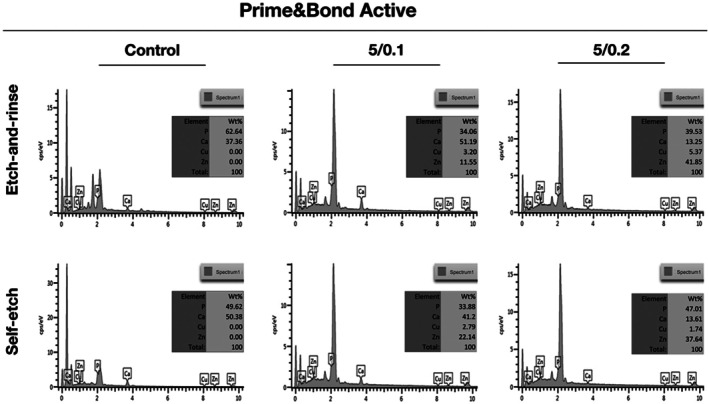
EDX spectrum of the resin–caries‐affected dentin interfaces after 2 years of water storage for 5/0.1% and 5/0.2% ZnO/CuNp‐containing Prime&Bond Active universal adhesive. The figure table summarizes the elemental composition of the sample outlined area.

**FIGURE 4:**
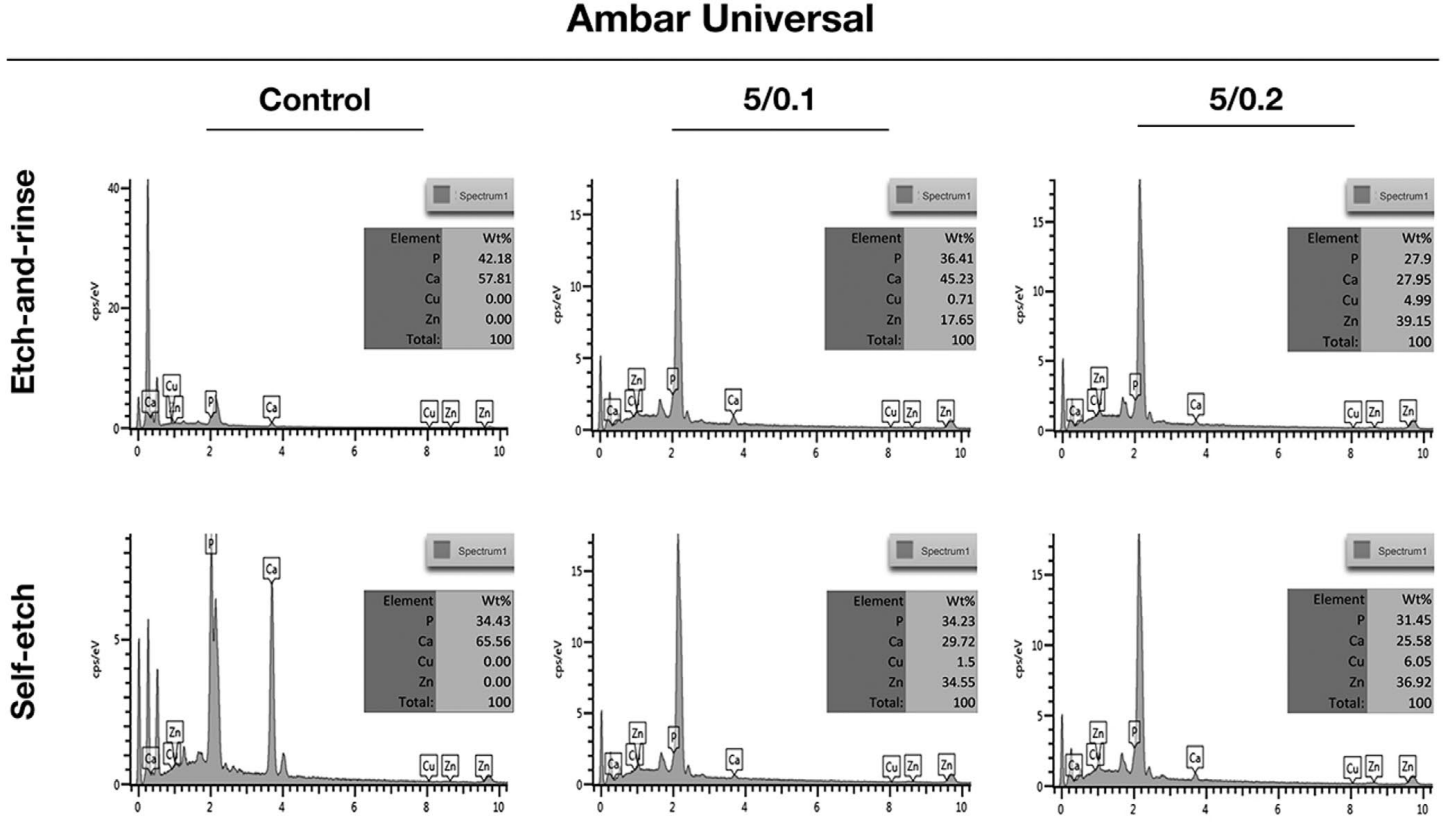
EDX spectrum of the resin–caries‐affected dentin interfaces after 2 years of water storage for 5/0.1% and 5/0.2% ZnO/CuNp‐containing Ambar Universal adhesive. The figure table summarizes the elemental composition of the sample outlined area.

The compositional analysis of the adhesive interface conducted by EDX after 2‐year revealed the presence of zinc and copper within the hybrid layer in both concentrations groups, across both adhesive strategies and adhesive systems (Figures 3 and 4). Also, the analysis showed the presence of calcium and phosphorus, which confirmed that the evaluation site was within the hybrid layer rather than limited to the adhesive layer (Figures 3 and 4).

## 4 Discussion

Most of the in vitro studies where experimental adhesive systems are tested do so on sound dentin, which is paradoxical considering that caries‐affected dentin is the most common substrate in daily clinical practice [1, 2, 41], especially when employing minimally invasive strategies that involve partial caries removal. The aims of this technique are to preserve tooth structure, minimizing the risk of injury to the pulp [5], and maintain the partially demineralized, remineralizable, and not yet colonized by bacteria zone of caries‐affected dentin [6, 8]. On the other hand, if we consider the studies that have been carried out on caries‐affected dentin, we can see that the vast majority only show short‐term results [1], and there are not many studies showing long‐term results on carious dentin.

Along these lines, a previous study showed that the incorporation of ZnONp and CuNp in concentrations up to 5/0.2 wt% in UAs improves the integrity of the hybrid layer on caries‐affected dentin at baseline [34]. However, the idea behind the incorporation of ZnONp and CuNp into adhesive formulations was to improve the long‐term bonding properties to caries‐affected dentin. Thus, this is the first study that was capable of demonstrating that the incorporation of ZnO/CuNps in two UAs improves the integrity of the adhesive interface on caries‐affected dentin, compared with ZnO/CuNps free adhesive, at the baseline and even after 2 years of water storage.

In this study, in terms of resin–caries‐affected dentin bond strength, we can observe that, for both UAs, no significant differences were observed among all groups at the baseline, regardless of adhesive strategies or addition of ZnO/CuNps, as well as previously observed for dentin bond strength in sound dentin [33]. However, for both UA systems, all ZnO/CuNps groups exhibited a significant decrease in NL values in resin–caries‐affected dentin for both adhesive strategies at the baseline when compared to control groups. Thus, the first research hypothesis was accepted. Several hypotheses may explain these NL results. Cu potentially enhances the strength of the altered organic matrix of the collagen fibrils by caries‐affected dentin, because lysyl oxidase (LOX), an enzyme responsible for collagen cross‐linking, is copper‐dependent [32]. This suggests copper may act indirectly as a cross‐linking agent. Taking into account that dentin in caries‐affected dentin is highly demineralized [6], studies have demonstrated that incorporating ZnO can lead to the formation of apatite crystallites on collagen fibrils, promoting dentin mineralization [35] and enhancing the cross‐linking effect. Also, ZnO has the ability to form zinc methacrylate and dimethacrylate, through the reaction with carbonyl, aromatic, N—H amide and acid groups of the adhesive, and to interact with urethane groups through the surface hydroxyl group, thus creating a polymer with strong internal interactions [42].

Moreover, considering the NL values observed even in the groups with ZnO/CuNps addition, it became evident that, despite the higher bond strength and lower NL achieved when UAs containing ZnO/CuNps were applied to caries‐affected dentin, some degree of degradation still occurred after 2 years. Thus, the second research hypothesis was accepted.

Although we know that this decrease in adhesive properties is consistent with the long‐term evaluation [43], the groups with ZnO and Cu always had statistically higher values of μTBS and statistically lower values of NL than control, irrespective of UA and adhesive strategies. Therefore, it's important that the ZnO/CuNps showed some additional action after water storage to maintain the preservation of the hybrid layer formed by caries‐affected dentin. Several mechanisms help to explain this “protective” effect when ZnO/CuNps are added into UAs. Studies have shown that caries‐affected dentin exhibits a greater rate of enzyme expression and/or activation compared to healthy dentin [12]. This is likely due to the intermittent pH changes that occur during caries progression, which can activate enzymes that degrade collagen [11].

Previous studies showed that, ZnO and Cu alone [23, 24, 25, 27, 31], or in combination inhibited MMP‐2, MMP‐8, and MMP‐9 [28], enzymes proteolytically active during any demineralization process, but they are still more abundant during tooth decay [10]. This inhibition could potentially help preserve the integrity of the dentin, the layer under the enamel, and slow down the progression of cavities.

These results over time can be partially related to the presence of ZnO/Cu within the hybrid layer even after 2 years of water storage. Thus, in this study we can observe the presence of ZnO/Cu within the hybrid layer in both concentrations, in both adhesive strategies and in both adhesive systems; therefore, this leads the authors to accept the third research hypothesis.

The presence of ZnO/Cu inside the resin–dentin adhesive interface after 2 years of water storage shows that the addition of ZnO/CuNps to UA systems could be used as a ZnO and Cu reservoir [33, 34]. Previous studies that evaluated the release of Cu showed that only 50% of the Cu added was released until about 1.3 years [17, 19]. This reservoir could help to a slow and constant controlled release of these nanoparticles around collagen fibrils in the hybrid layer. That permits a sustained release could keep ZnO and Cu at a slow concentration on the adhesive interface for a long time and be capable of maintaining and/or retarding the bonding degradation in the oral interface, on a challenging dentin such as caries‐affected dentin. Thus, this may be a way to extend the life expectancy of adhesive restorations on carious dentin.

While the constant and prolonged release of Zn and Cu ions is important for maintaining the longevity of the hybrid layer, it must be accompanied by low cytotoxicity and good biocompatibility of both the UAs and the incorporated ions. In this regard, a recent study demonstrated that, for Prime&Bond Active UA, incorporating ZnO/CuNps at the concentrations tested in this study increased cytotoxicity compared with the commercial adhesive [33]. This effect was attributed to a deficient chemical interaction between the nanoparticles and the adhesive components, which increased water sorption and, consequently, the elution of inadequately polymerized monomers and other resin components along with the nanoparticles. In contrast, for Ambar Universal, no significant differences were observed between the ZnO/CuNps concentrations and the control (commercial material), likely because groups 5/0.1 and 5/0.2 exhibited low water sorption and solubility compared with the control [33]. This suggests that the nanoparticles were not released as quickly, thereby avoiding a cytotoxic effect.

The present study was capable of demonstrating that the addition of ZnO/CuNps into two UAs systems improves the quality of resin–caries‐affected dentin interface, decreasing and retarding hybrid layer degradation after 2 years. Although this is the first study to show the effect of adding ZnO/CuNps on the medium‐term bonding performance of UA on caries‐affected dentin, the present results are in agreement with previous findings on sound dentin [17, 18, 20, 28]. However, since only clinical studies have evaluated the incorporation of CuNps in UAs [21, 22], future research, such as in situ or additional clinical studies, is needed to investigate the effects of adding ZnO/CuNp on UAs.

A limitation of this study that must be acknowledged is its laboratory‐based design, which does not allow direct extrapolation of the results to clinical practice. However, the prolonged evaluation time provides strong in vitro evidence supporting the favorable effects of zinc oxide and copper nanoparticles in the hybrid layer stability over time.

## 5 Conclusion

The incorporation of zinc oxide and copper nanoparticles at concentrations up to 5/0.2 wt% into two UA systems reduced NL and preserved the μTBS to caries‐affected dentin, likely due to their sustained presence of these ions within the hybrid layer even after 2 years. Thus, incorporating ZnO/CuNp into UAs may enhance the stability of adhesive restorations on caries‐affected dentin by minimizing interfacial degradation.

## Funding

This project was supported by Conselho Nacional de Desenvolvimento Científico e Tecnológico, 304444/2025‐1; Agencia Nacional de Investigación y Desarrollo 11221070 and 1170575; Coordenação de Aperfeiçoamento de Pessoal de Nível Superior, 001.

## Conflicts of Interest

The authors declare no conflicts of interest.

## Data Availability

The data that support the findings of this study are available on request from the corresponding author. The data are not publicly available due to privacy or ethical restrictions.
